# Cannabinoid Hyperemesis Syndrome: Case Report of a Paradoxical Reaction with Heavy Marijuana Use

**DOI:** 10.1155/2012/757696

**Published:** 2012-05-28

**Authors:** Benjamin Cox, Akansha Chhabra, Michael Adler, Justin Simmons, Diana Randlett

**Affiliations:** Department of Internal Medicine, New York University School of Medicine, 550 First Avenue, New York, NY 10016, USA

## Abstract

Cannabinoid hyperemesis syndrome (CHS) is a rare constellation of clinical findings that includes a history of chronic heavy marijuana use, severe abdominal pain, unrelenting nausea, and intractable vomiting. A striking component of this history includes the use of hot showers or long baths that help to alleviate these symptoms. This is an underrecognized syndrome that can lead to expensive and unrevealing workups and can leave patients self-medicating their nausea and vomiting with the very substance that is causing their symptoms. Long-term treatment of CHS is abstinence from marijuana use—but the acute symptomatic treatment of CHS has been a struggle for many clinicians. Many standard medications used for the symptomatic treatment of CHS (including ondansetron, promethazine, and morphine) have repeatedly been shown to be ineffective. Here we present the use of lorazepam as an agent that successfully and safely treats the tenacious symptoms of CHS. Additionally, we build upon existing hypotheses for the pathogenesis of CHS to try to explain why a substance that has been used for thousands of years is only now beginning to cause this paradoxical hyperemesis syndrome.

## 1. Introduction

Marijuana is a popular recreational drug that comes from the *Cannabis* genus of flowering plants. It also has several medical applications—two of the most recognized being its use as an antiemetic and an appetite stimulant [1, 2]. However, heavy prolonged use of this drug has been shown to cause the poorly-understood, paradoxical cannabinoid hyperemesis syndrome (CHS). Cannabinoid hyperemesis syndrome was first described by Allen et al. in 2004 [3]. Sontineni et al. described diagnostic criteria that include: essential feature of chronic cannabis use; major features of recurrent severe nausea and vomiting that resolves after stopping cannabis use; supportive features of compulsive hot baths with symptom relief, colicky abdominal pain, and no evidence of gall bladder or pancreatic inflammation [4].

## 2. Case Presentation

A 28-year-old man with no past medical history presented to the emergency department with a two-week history of severe “10-out-of-10” colicky epigastric pain with profound nausea, 15–20 episodes of vomiting daily, and decreased oral intake for fear of triggering these symptoms. He denied alcohol and tobacco use, but reported he had smoked two marijuana cigars (each containing approximately 1.5 grams marijuana bud) every day for the last ten years. As his symptoms of nausea, vomiting, and abdominal pain intensified, he self-medicated himself with increasing amounts of marijuana and his symptoms became increasingly intense. The patient reported that he initially got symptomatic relief with a hot shower, though as his symptoms intensified, he required increasingly longer bathing times that eventually progressed to the patient soaking himself in hot baths for hours each day. He stated that he had never experienced any of these symptoms in the past. In the emergency department, the patient's vital signs were within normal limits and his physical exam was significant only for minor tenderness to palpation in the epigastric region. The patient had no focal neurological deficits. The patient was admitted for intravenous fluid support, antiemetic therapy, and further evaluation.

The patient's medical evaluation revealed a complete blood count, basic metabolic profile, and hepatic profile that were within normal limits. An abdominal computerized tomography (CT) scan was negative for pathology, an EGD with biopsies showed only mild gastritis, and a gastric emptying study showed mildly delayed gastric emptying.

In this patient, initial symptomatic treatment with ondansetron and morphine was unable to keep the patient from having breakthrough episodes of nausea, vomiting, and epigastric pain. The patient was unable to tolerate even a clear liquid diet, and was extremely anxious about trying to increase or advance his oral intake given the painful episodes that food had triggered in the past. After administering 1 mg IV lorazepam, the patient improved at a remarkable pace; within 10 minutes of administration he no longer experienced nausea, abdominal, or food aversion. Over the next 12 hours, he transitioned to a regular diet, oral lorazepam (1 mg tablets), and was able to discontinue all other analgesic and antiemetic medications. The time from marijuana cessation to complete resolution of symptoms was approximately three weeks. The patient was discharged with a seven-day prescription for lorazepam (1 mg PO, twice daily). The patient was contacted at 3 and 6 months after discharge and he reported that, with sustained abstinence from marijuana, he had no return of his symptoms.

## 3. Discussion and Conclusions

It has been hypothesized that the principal psychoactive constituent of the cannabis plant, Delta-9-tetrahydrocannabinol (THC) is the causative agent in CHS [3]. THC has been shown to cause delayed gastric emptying and thermoregulatory disturbances via action on the cannabinoid receptor type 1 (CB1) in the enteric plexus and central nervous system, respectively [5]. Chronic stimulation of the CB1 receptors may result in the development of the gastrointestinal and thermodysregulation symptoms in sensitive patients [6], though more research needs to be conducted to further elucidate what factors may predispose certain patients.

The earliest recorded uses of marijuana date from the 3rd millennium BC [7], so why are we only now beginning to see patients with CHS? We hypothesize that the emergence of this syndrome is related to changes in patterns of plant component utilization, and technological advances in the production of marijuana that have dramatically increased THC concentrations. In past decades, the stalks, leaves, and buds of both male and female plants were consumed. Modern analysis of the cannabis plant shows that the buds (flowers) of the female cannabis plant contain the highest concentrations of THC and that the leaves can contain ten times less THC than the buds, and the stalks one hundred times less THC [8]. This new understanding has led to the virtual elimination of stalks and leaves from consumption; now modern users almost exclusively buy and consume the highly-potent female buds and thus consume significantly higher amounts of THC. Furthermore, advances in breeding and cultivation techniques, including the use of hydroponics, cloning, high-intensity artificial lighting, and optimization of growing conditions (e.g., ambient temperature and air gas concentrations) have increased the potency of modern cannabis. Recent research undertaken at the University of Mississippi's Potency Monitoring Project has found that the mean THC content in confiscated cannabis samples increased from 3.4% in 1993 to 8.8% in 2008 [9]. We believe that the modern consumption of higher-potency preparations of higher-potency marijuana is causing profoundly higher exposure to patients to THC and may be contributing to the emergence of the cannabinoid hyperemesis syndrome.

Anticipatory nausea and vomiting is seen in patients undergoing chemotherapy and lorazepam's antiemetic, amnestic, and anxiolytic properties have made it helpful for treatment of this condition [10]. Lorazepam has also been suggested in the treatment of cyclic vomiting syndrome (CVS) [11]. One case report notes that benzodiazepines were given for one week to a patient with CHS, but the patient continued cannabis use, possibly limiting their efficacy [12]. We found that lorazepam was extremely effective in treating our patient's CHS. It helped the patient overcome his conditioning to see food as a painful emetic trigger and also helped to ameliorate the physical symptoms of CHS. The addition of lorazepam to the patient's regimen allowed for rapid transition from a clear liquid to a regular diet, and the ability to tolerate oral medications. We recommend that lorazepam be considered in the treatment of CHS, especially in patients with strongly conditioned food aversion.

It is unclear how this substance used by many to treat nausea and stimulate appetite causes a condition with severe nausea, vomiting, and anorexia with food anxiety. This lack of understanding and delayed diagnosis can lead patients to self-medicate with the very substance that is causing their suffering. As such, patient education is critical. For providers, increased awareness of this syndrome and its inclusion in the differential diagnosis of patients with intractable nausea and vomiting may help prevent expensive and unrevealing workups and decrease patient morbidity. Because many patients with CHS have been chronic, heavy marijuana users, it is important to assess if/how they may have incorporated their substance use into their social lives and daily routines. A thoughtful discussion about abstinence strategies, and referral for mental health support and chemical dependence treatment may also benefit patients with cannabinoid hyperemesis syndrome.
